# Early rapidly developing constrictive pericarditis after aortic valve surgery

**DOI:** 10.1007/s12471-013-0514-8

**Published:** 2014-01-09

**Authors:** J. J. Brugts, A. Constantinescu, A. P. Kappetein, S. W. E. van de Poll, K. Caliskan, O. C. Manintveld

**Affiliations:** 1 Department of Cardiology (Heart Failure, Transplantation & Mechanical Circulatory Support Unit), Erasmus MC, Thoraxcenter, ‘s Gravendijkwal 230, 3015 CE Rotterdam, the Netherlands; 2 Department of Cardiothoracic Surgery, Erasmus MC, Thoraxcenter, ‘s Gravendijkwal 230, 3015 CE Rotterdam, the Netherlands; 3 St Franciscus gasthuis, Rotterdam, the Netherlands

## Case report

**Fig. 1:**
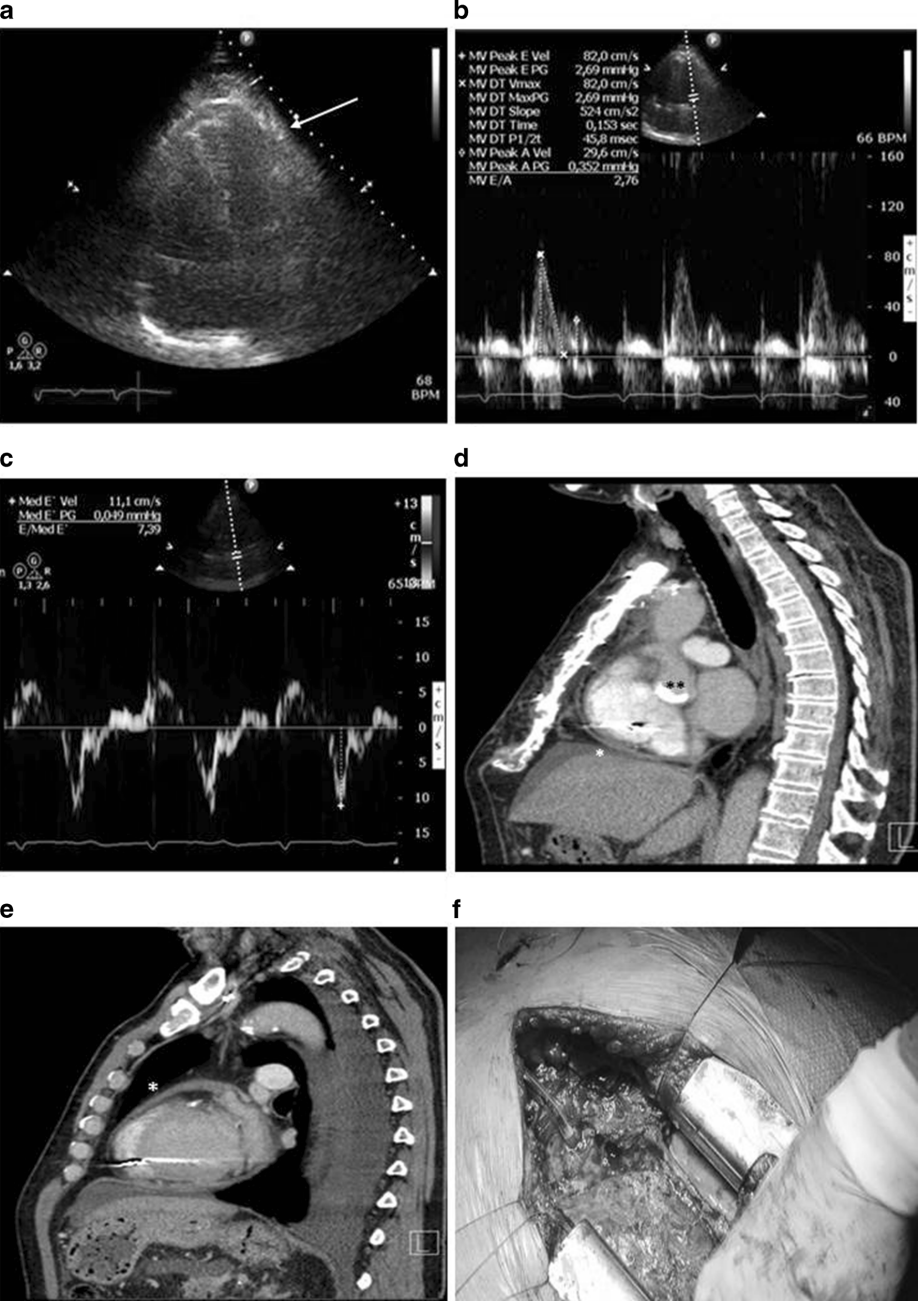
**a**. Transthoracic echocardiographic image of apical four-chamber view, demonstrating normal LV function with an undefined echodense layer or mass at LV apex **b**. Transthoracic echocardiographic image of transmitral inflow pattern using pulsed wave Doppler imaging **c**. Transthoracic echocardiographic image of mitral annual velocity using tissue Doppler imaging of septal mitral valve annulus movement demonstrating normal LV fillings pressure (E/E’ ratio of 7.4) **d**. CT image of the pericardium showing thickened pericardium without calcifications by *single asterisk* and the On-X aortic valve by *double asterisk* **e**. CT image of the pericardium showing thickened pericardium without calcifications by *single asterisk* **f**. Perioperative photography during pericardiectomy revealing fibrotic, thickened and organised pericardium. Histological examination (PA) confirmed the diagnosis of constrictive pericarditis

**Fig. 2:**
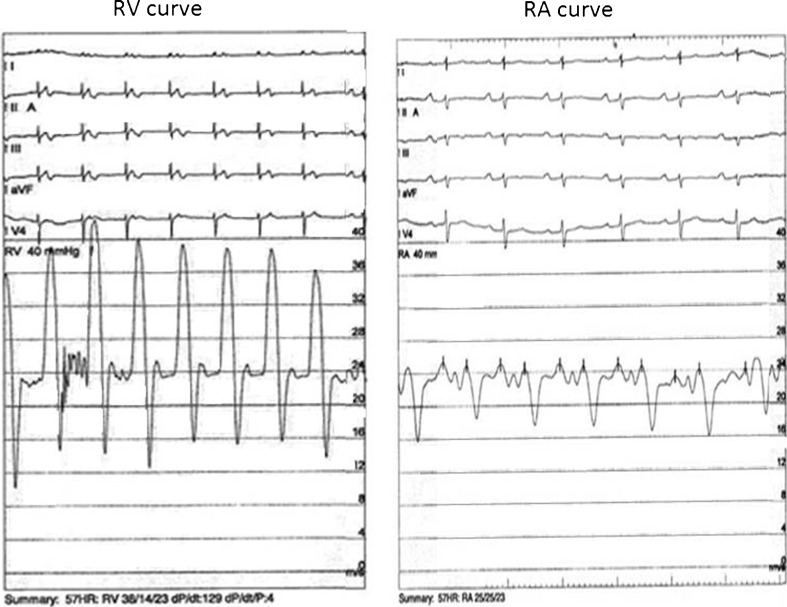
Right heart catheterisation revealed a diastolic dip plateau phase in the right ventricle pressure waves (figure **a**, RV curve) and right atrial Y-wave (figure **b**, RA curve) suggestive of constrictive pericarditis

A 58-year-old male patient underwent aortic valve replacement surgery (On-X mechanical prosthesis, ring size 23 mm) due to severe aortic valve stenosis with preserved LV function and normal coronary arteries. The postoperative period was complicated by third-degree AV block and sustained ventricular tachycardias and a DDD-ICD implantation was implanted. After 3 months of surgery, the patient presented with symptoms of progressive fatigue, dyspnoea on exertion and generalised peripheral oedema. No chest pain was reported. The patient had gained 30 kg in weight in 3 months. Physical examination revealed a raised jugular venous pressure with prominent vein pulsations, normal cardiac sounds with normal artificial valve clicks and no murmurs. Auscultation of the lungs was normal. The patient had a palpable liver of 4 cm, noticeable ascites, and extensive pitting oedema from ankle to hip. Laboratory results revealed elevated liver enzymes and moderately raised NT-proBNP (328 pmol/l) without other abnormalities. Electrocardiogram revealed a sinus rhythm with ventricular pacing. Chest X-ray demonstrated an enlarged heart but no pulmonary congestion. The echocardiogram was limited by severely impaired image quality. Left ventricular function was normal, right heart function was mildly impaired and LV diastolic function was normal with E/A ratio 2.7 (E 0.82; A 0.30; deceleration time 153 ms), with normal E/E’ ratio 7.4 (E’ = 11.1) (Fig. 1a–c). The mechanical aortic valve was sufficient. Respiratory variation of transmitral flow velocity signal appeared >25 % but not conclusive because of limited interpretability due to low echo quality. During echocardiography, an echodense layer or mass was seen at the LV apex outside the myocardium, which could be pericardial fluid (blood or thrombus), thickened pericardium or epicardial fat. A chest CT proved the layer to be a thickened pericardium (6 mm) (Fig. 1d, e). Although unlikely so soon after surgery, the signs of previously absent right-sided heart failure and the thick pericardium suggested the diagnosis of constrictive pericarditis without any signs of calcification yet. Other causes such as inflammatory (normal CRP) or autoimmune disorders (M-protein negative) were ruled out. Differential diagnostics now included constrictive pericarditis and restrictive cardiomyopathy. The normal E’ of 11.1 makes restrictive cardiomyopathy unlikely (in which the myocardium itself is diseased). To confirm the diagnosis of constrictive pericarditis (which would have severe consequences for the patient as a re-thoracotomy would be necessary), we performed a right heart catheterisation. The right heart catheterisation revealed a diastolic dip plateau phase (Fig. 2a) and right atrial Y wave further confirming our diagnosis of constrictive pericarditis (Fig. 2b). We did not perform simultaneous left-sided pressure measurements with the mechanoprothesis in situ. At that stage, we discussed re-operation with the cardiothoracic surgeon and decided to perform pericardiectomy. Surgery revealed a thickened pericardium and an extensive fibrous net/rind of the thickened pericardium was found and removed (Fig. 1f). Histological examination of the removed material confirmed the diagnosis with signs of chronic fibrotic inflammation. After pericardiectomy, the patient recovered slowly with a regimen of intravenous diuretics and positive inotropes (enoximone) for the first postoperative days. He was discharged in a good condition on oral diuretics.

## Discussion

Constrictive pericarditis (CP) is a rare complication after cardiac surgery, which is infrequently reported in the literature [1–3]. Constrictive pericarditis may develop as a midterm or late complication of cardiac surgery [1]. The incidence is estimated at 0.2–2.4 % [1]; however, the short-term occurrence as in our case has not been described earlier. It is a disease characterised by the encasement of the heart by a rigid non-pliable pericardium due to dense fibrosis and adhesions. This causes impaired cardiac filling, exaggerated by the enhanced interventricular interdependence, leading to heart failure manifested by right-sided congestion (by impaired filling), peripheral oedema and ascites, besides symptoms of diminished cardiac output in response to exertion with fatigability and dyspnoea on exertion. The current case is unique in the early and rapid development of constrictive pericarditis within 3 months after cardiac surgery, which is reported in the literature as a complication occurring 1.5–2 years after surgery [4]. The treatment for constrictive pericarditis is radical pericardiectomy, which is a high-risk procedure with a high incidence of complications and mortality but in most patients symptoms are relieved [5, 6]. Recurrence of constrictive pericarditis is possible, but the recurrence rate is not exactly known [5, 6]. Recurrent right-sided heart failure after operation for constrictive pericarditis may be caused by incomplete pericardiectomy, or recurrent constriction due to exuberant scar tissue [5, 6]. Recently, colchicine was reported to be a safe and effective treatment to prevent postcardiectomy syndrome [7] and can probably prevent the late complications such as constrictive pericarditis. Although rare, cardiologists and cardiothoracic surgeons should be wary of this clinical problem and rapid diagnosis and treatment of constrictive pericarditis are crucial to reduce mortality and morbidity. Pericardiectomy should be performed early after diagnosis, in order to prevent chronic illness. After surgery, inotropes, diuretics, salt restriction, and nutrition supply are also critical to improve the prognosis of the patient [8].
